# ASO Author Reflections: Reassuring Timeliness in the Era of Second Opinions

**DOI:** 10.1245/s10434-025-18115-w

**Published:** 2025-08-20

**Authors:** Pooja Varman

**Affiliations:** Cleveland Clinic Foundation, 9500 Euclid Avenue, Cleveland, OH 44195 USA

## Past

Patients often seek second opinions after a breast cancer diagnosis to confirm findings, explore treatment options, or gain trust in their care plan.^1^ While these consultations can refine or even alter clinical management, they also raise concerns about potential treatment delays.^2, 3^ National guidelines recommend breast surgery within 60 days of diagnosis for early-stage breast cancer, but whether second-opinion evaluations meaningfully delay care remains uncertain. Prior studies have demonstrated variable delays associated with transferring care,^4^ yet little is known about how time to treatment compares once externally diagnosed patients initiate care at a comprehensive cancer center.

## Present

In our study of 226 patients with stage 0–III breast cancer, we found that those diagnosed externally and seeking a second opinion at our center experienced a modest delay—10 days longer from biopsy to treatment—compared with those diagnosed internally. This difference was largely driven by the need for additional imaging and biopsies, particularly among externally diagnosed patients. However, once patients established care within our institution, there was no difference in time from surgical consultation to treatment, and both groups were treated within the 60-day benchmark. These findings are reassuring for patients and providers alike, reinforcing that seeking second opinions for early-stage breast cancer need not compromise timely care.^5, 6^

## Future

Efforts should focus on streamlining diagnostic work-up and referral pathways for externally diagnosed patients. Standardizing image-sharing protocols and fostering inter-institutional collaboration may help reduce avoidable delays. Multi-institutional studies are needed to assess whether similar patterns hold across other cancer centers and to identify scalable interventions that promote both timely and personalized care for patients navigating second opinions.
